# Curcumin Modulates the Crosstalk Between Macrophages and Bone Mesenchymal Stem Cells to Ameliorate Osteogenesis

**DOI:** 10.3389/fcell.2021.634650

**Published:** 2021-02-09

**Authors:** Songfeng Chen, Hang Liang, Yanhui Ji, Hongwei Kou, Chi Zhang, Guowei Shang, Chunfeng Shang, Zongmian Song, Lin Yang, Lei Liu, Yongkui Wang, Hongjian Liu

**Affiliations:** ^1^Department of Orthopaedics, The First Affiliated Hospital of Zhengzhou University, Zhengzhou, China; ^2^Department of Orthopaedics, Union Hospital, Tongji Medical College, Huazhong University of Science and Technology, Wuhan, China; ^3^Department of Paediatrics, The Zhengzhou Central Hospital Affiliated to Zhengzhou University, Zhengzhou, China

**Keywords:** curcumin, osteogenesis, immune modulation, macrophages, inflammatory microenvironment

## Abstract

Bone healing is thought to be influenced by the cross-talk between bone forming and immune cells. In particular, macrophages play a crucial role in the regulation of osteogenesis. Curcumin, the major bioactive polyphenolic ingredient of turmeric, has been shown to regulate inflammatory response and osteogenic activities. However, whether curcumin could regulate macrophage polarization and subsequently influence osteogenesis remain to be elucidated. In this study, the potential immunomodulatory capability of curcumin on inflammatory response and phenotype switch of macrophages and the subsequent impact on osteogenic differentiation of MSCs are investigated. We demonstrated that curcumin exhibited significant anti-inflammatory effect by polarizing the macrophages toward anti-inflammatory phenotype, with increased expression of IL-4, IL-10, and CD206, and decreased expression of IL-1β, TNF-α, CCR7, and iNOS. In addition, curcumin could improve the osteo-immune microenvironment *via* promoting osteogenesis-related regenerative cytokine BMP-2 and TGF-β production. Moreover, the co-cultured test of macrophages and BMSCs showed that curcumin-modulated macrophages conditioned medium could promote osteogenic differentiation of BMSCs with increased gene (ALP, Runx-2, OCN, and OPN) and protein (Runx-2 and OCN) expression levels, enhanced ALP activity, and obvious formation of mineralized nodules. Taken together, with the interaction between curcumin-conditioned macrophage and curcumin-stimulated BMSCs, curcumin could remarkably enhance the osteogenic differentiation of BMSCs in LPS-activated inflammatory macrophage-BMSCs coculture system.

## Introduction

Macrophages have been shown to play an essential role in innate immunity and maintain homeostasis and tissue regeneration (Sica et al., 2015). The resident macrophages in most tissues can efficiently and rapidly adapt to the dynamic microenvironment changes in order to modulate critical tissue-specific functions (Park and Barbul, 2004; van Amerongen et al., 2007). After a bone injury, as the first line of host defense against microorganisms, macrophages are recruited from peripheral blood and bone marrow and increase greatly (Alexander et al., 2011; Vi et al., 2015). Throughout the process of tissue regeneration, macrophages exhibit tremendous plasticity and can adapt various intermediate states from pro-inflammatory M1 phenotype to anti-inflammatory M2 phenotype. M2 phenotype macrophages include subtypes such as M2a, M2b, M2c, and M2d. These subtypes play relevant but distinct roles during the normal healing process. In this study, we use the terms “M1 macrophages” and “M2 macrophages” to describe macrophages that exhibit divergent effects of pro-inflammation and anti-inflammation. During the period of bone repair, uncommitted macrophages (M0) become polarized toward an appropriate phenotype to regulate the healing process (Brown et al., 2012). During the acute inflammation period, macrophages exhibit a pro-inflammatory phenotype (M1) to defend against the invasion of pathogens and restore tissue homeostasis. At later stages of healing, M1 macrophages switch to the anti-inflammatory phenotype (M2) to promote bone repair by producing growth factors to recruit progenitor cells and guide the osteo-differentiation. Stalled M1-to-M2 transition was found to be detrimental to bone healing (Ho and Sly, 2009; Guihard et al., 2012). In recent years, research has determined that the modulation of the cross-talk between bone forming cells and macrophages and bone precursor cells was critical for bone healing (Walsh et al., 2006; Guder et al., 2020).

MSCs derived from bone marrow were able to develop into bone-forming cells *in vitro* and regenerate bone tissue *in vivo*. They are thought to be a reliable source for bone regenerative medicine (Keating, 2006; Uccelli et al., 2008). Besides, MSCs were found to have wide immunomodulatory capability, making them attractive targets for tissue engineering applications (Abumaree et al., 2013). Curcumin, a major bioactive compound that is mainly extracted from the root of turmeric (Curcuma longa), exhibits multiple pharmacological properties such as anti-oxidant, anti-inflammatory, anti-carcinogenic, and neurotrophic properties (Hussain et al., 2017; Li W. et al., 2017; Zhang et al., 2019). Several studies have demonstrated the beneficial role of curcumin in bone disorders and inflammatory diseases, including osteolysis, periodontitis, rheumatoid arthritis, and osteoporosis (Hie and Yamazaki, 2009; Martins et al., 2015). In addition, previous studies have revealed that curcumin could polarize macrophages to an anti-inflammatory phenotype to suppress inflammation through Toll-like receptor 4 (TLR4)-mediated signaling pathway, avoiding the chronic inflammation at the injury site (Zhou and Zhang, 2015; Zhou et al., 2015). Moreover, curcumin could enhance osteogenic differentiation of stem cells and inhibit osteoclast resorption through PI3K/AKT/Nrf2 signaling pathway (Kim et al., 2012; Chen et al., 2014). However, the exact regulatory mechanism of the crosstalk between macrophages and BMSCs and the subsequent effects on bone tissue regeneration are crucial for the application of curcumin in bone regenerative medicine. Whether and how curcumin participate in osteo-immunomodulation in the bone healing process remained unknown. Thus, further studies are still needed to investigate the modulatory effects of curcumin on bone injury disease.

In this study, we investigate the influence of curcumin on macrophage polarization and inflammatory response and subsequent effects on osteogenic differentiation of BMSCs. We focus on the regulatory role of curcumin in coordinating the crosstalk between macrophages and BMSCs. Then the macrophage-BMSCs coculture system was used to explore the exact mechanisms of curcumin-mediated osteo-immunomodulation for bone regeneration.

## Materials and Methods

### Reagents

Curcumin and lipopolysaccharide (LPS, *Escherichia coli O127:B8*; purity 99%) were purchased from Sigma-Aldrich (United States). Dulbecco’s modified eagle medium (DMEM) and fetal bovine serum (FBS) were purchased from Gibco (United States). Alkaline Phosphatase (ALP) Assay Kit was purchased from Beyotime (Shanghai, China). Alizarin Red S was purchased from Cyagen (United States). Enzyme-linked immunosorbent assay (ELISA) kits were purchased from Boster (Wuhan, China). The primary antibodies for runt-related transcription factor 2 (Runx-2), osteocalcin (OCN), and glyceraldehyde-3-phosphate dehydrogenase (GAPDH) were purchased from Cell Signaling Technology, Inc. (United States). The secondary antibodies (horseradish peroxidase-conjugated anti-rabbit immunoglobulin G) were obtained from Santa Cruz Biotechnology (Santa Cruz, CA, United States). The fluorescence secondary antibody for TRITC (Tetramethyl Rhodamin Isothiocyanate) AffiniPure Goat Anti-Rabbit IgG was purchased from EarthOx (San Francisco, CA, United States). The DAPI (4′,6-Diamidino-2-Phenylindole) Staining Kit was obtained from Boster (Wuhan, China). Antibody specific for flow cytometry, including CCR7-PE (phycoerythrin) and CD206-APC (allophycocyanin), were purchased from Biolegend (San Diego, CA, United States).

### Cell Culture

All experimental protocols were approved by the Ethical Committee for Animal Experiments of The First Affiliated Hospital of Zhengzhou University. Murine macrophage RAW 264.7 cells and BMSCs derived from murine bone marrow were purchased from China Center for Type Culture Collection. The cells were cultured in Dulbecco’s Modified Eagle’s Medium (DMED, Hyclone, United States) supplemented with 10% FBS (Gibco) and 1% penicillin-streptomycin solution (Gibco) in an atmosphere of 5% CO_2_ at 37°C. The cells were passaged at approximately 80% confluence and used at early passages (p3–5).

### Effects of Curcumin on Macrophage Polarization and Inflammatory Response

In this study, *E. coli* lipopolysaccharide was used as the inflammation stimulus to investigate the effects of curcumin on the inflammatory response and phenotype switch of macrophages. To investigate the effects of curcumin on macrophage polarization and inflammatory response in non-inflammatory environment, RAW 264.7 cells (5 × 10^5^ cells/well) were cultured in 24-well plates with Dulbecco’s Modified Eagle’s Medium containing 10% FBS overnight at 37°C and pre-treated with PBS or curcumin with various dosages (5, 10, and 20 μmol/L) for 6 h for further experiment. Then, to investigate the regulatory role of curcumin in inflammatory environment, the cells were incubated in the presence or absence of LPS (20 ng/mL) for 12 h.

#### Flow Cytometry

Briefly, RAW 264.7 cells were digested and blocked with CD16/32 for 5 min at 4°C. Then the cells were incubated with PE-conjugated CCR7 (BioLegend) and APC-conjugated CD206 (BioLegend) for 30 min at 4°C. After that, cells were analyzed by a flow cytometer (BD FACSCalibur). The data were analyzed by FlowJo software.

#### Enzyme-Linked Immunosorbent Assay

After RAW 264.7 cells were stimulated to polarize, the supernatant was collected and centrifuged at 3,000 rpm/min for 10 min. ELISA kit (Boster, China) was used to detect the concentration of TNF-α, IL-1β, IL-4, and IL-10 following the manufacturer’s instructions.

#### Immunofluorescence Staining

RAW 264.7 cells in different groups were fixed in 4% paraformaldehyde, permeabilized with 0.25% Triton-X, and blocked by 1% BSA. Subsequently, the cells were incubated with the primary antibodies against iNOS and CD206 with 1:100 dilution overnight at 4°C. Then the cells were incubated with the respective fluorescein secondary antibody for 30 min, followed by 5 min of nuclear staining with DAPI. The samples were observed with a Laser Scanning Confocal Microscopy (LSCM).

#### RNA Preparation and Quantitative Real-Time Polymerase Chain Reaction

The method of quantitative real-time polymerase chain reaction (qRT-PCR) was described previously (Hu et al., 2020). Total RNA was obtained by TRIzol Reagent (Thermo Fisher Scientific, United States) according to the manufacturer’s instructions. Complementary DNA was prepared from the isolated RNA by PrimeScript RT Reagent Kit (Takara Bio, Otsu, Japan). Finally, real-time PCR was performed on the Step One Plus Real-Time PCR system (Thermo Fisher Scientific, United States). The primers for qRT-PCR, including GAPDH, IL-4, IL-10, TNF-α, IL-1β, BMP-2, TGF-β, ALP, Runx-2, OCN, and OPN, were synthesized by Genscript (China). Primers are listed in Table 1. Relative quantification was achieved using the comparative 2^–ΔΔ*Ct*^ method.

**TABLE 1:** Primer sequences.

Gene	Forward primer sequences (5′–3′)	Reverse primer sequences (5′–3′)
IL-1β	TGCCACCTTTTGACAGTGATG	AAGGTCCACGGGAAAGACAC
TNF-α	CCCTCACACTCAGATCATCTTCT	GCTACGACGTGGGCTACAG
IL-4	CCATATCCACGGATGCGACA	AAGCCCGAAAGAGTCTCTGC
IL-10	GCTCTTACTGACTGGCATGAG	CGCAGCTCTAGGAGCATGTG
BMP-2	CAGCCCGATCACCTCTCTTC	GAGACCGCAGTCCGTCTAAG
TGF-β	TCGGGGCTGCGGCTACT	ACAGGATCTGGCCACGGA
ALP	GGACCATTCCCACGTCTTCAC	CCTTGTAGCCAGGCCCATTG
Runx-2	CGCCTCACAAACAACCACAG	GGTAGTGACCTGCGGAGATT
OCN	TCACACTCCTCGCCCTATTG	GGGTCTCTTCACTACCTCGC
OPN	GAGCGAGGATTCTGTGGA	TCGACTGTAGGGACGATTG
GAPDH	ACAACTTTGGTATCGTGGAAGG	GCCATCACGCCACAGTTTC

### Osteogenic Differentiation Evaluation of Macrophage-Conditioned Medium

After the RAW 264.7 cells were treated with or without the curcumin for 6 h and subsequently incubated with or without LPS for 12 h as above, the medium was collected and centrifuged at 14,000 rpm for 30 min. Then the acquired supernatant was mixed with normal osteogenic medium at a ratio of 1:2 to obtain the conditioned medium (CM) for further experiments. BMSCs cultured in normal culture medium were used as normal control (NC). BMSCs were cultured in 12-well plates at a density of 1 × 10^5^/well in DMEM containing 10% FBS overnight and then incubated in conditioned medium for osteogenic evaluation. The conditioned medium was replaced twice a week. First, to evaluate the effects of macrophage-related immune microenvironment on osteogenic differentiation of BMSCs, BMSCs were divided into five groups (NC group: BMSCs cultured in normal osteogenic medium; Ctrl group: BMSCs cultured in untreated-macrophage CM; Cur group: BMSCs cultured in curcumin-treated (20 μmol/L) macrophage CM; LPS group: BMSCs cultured in LPS-treated (20 ng/mL) macrophage CM; LPS + Cur group: BMSCs cultured in CM of macrophages treated with curcumin and LPS). Then, to investigate the direct effects of curcumin on BMSCs in inflammatory microenvironment, BMSCs were pre-treated with curcumin (20 μmol/L) for 6 h and then incubated in the LPS-treated macrophage CM. Finally, to comprehensively estimate whether curcumin could promote osteogenic differentiation of BMSCs under inflammatory condition *in vitro*, we combined the two interrelated and indispensable effects of curcumin on macrophages and BMSCs.

#### Alkaline Phosphatase Activity

Alkaline phosphatase activity of BMSCs was detected by ALP staining after the cells were cultured with different CM for 1 week. Briefly, the cells were fixed for 15 min and washed with PBS. Then the cells were incubated in ALP stain solution (BCIP/NBT ALP color development kit, Beyotime, China) for 30 min and then visualized with an inverted light microscope.

#### Mineralization

Mineralization of BMSCs was analyzed using Alizarin Red S (ARS) staining. The BMSCs were cultured for 14 days. Then the cells were fixed and stained with 1% ARS (Cyagen Biosciences Inc., United States) solution. Afterward, the staining images were captured with an inverted light microscope.

#### Osteogenesis-Related Gene Expression

BMSCs were cultured with corresponding CM in a 6-well plate (2 × 10^5^ cells/well) for 3 and 7 days. At each time point, total RNA was harvested for the RT-PCR detection as described in section “RNA Preparation and Quantitative Real-Time Polymerase Chain Reaction.”

#### Osteogenesis-Related Protein Expression of BMSCs

The method of western blot was described previously (Chen S.F. et al., 2017). Total proteins in cell lysates were extracted with a lysis solution, electrophoresed, and then transferred to polyvinylidene difluoride membranes. After blocked in 5% non-fat milk, Runx-2 (1:1000, Abcam), OCN (1:1,000, Abcam), and GAPDH (1:2,000, Affinity Biosciences) antibody were dropped onto the membranes and incubated overnight at 4°C. Then, the membranes were treated with corresponding secondary antibody. Finally, the protein bands were detected by enhanced chemiluminescence reagent system (Affinity Biosciences). The relative density was measured using ImageJ 1.37v software.

### Statistical Analysis

All experiments were performed three times. All the data were shown as the mean ± standard deviation (SD). GraphPad Prism 6.0 software was used to perform the statistical analysis. Differences between groups were analyzed using one-way analysis of variance (ANOVA) followed by the Tukey *post hoc* test. Differences were considered as statistically significant when *P* < 0.05.

## Results

### The Effects of Curcumin on the Regulation of Inflammatory Response and Macrophages Phenotype

**FIGURE 1:**
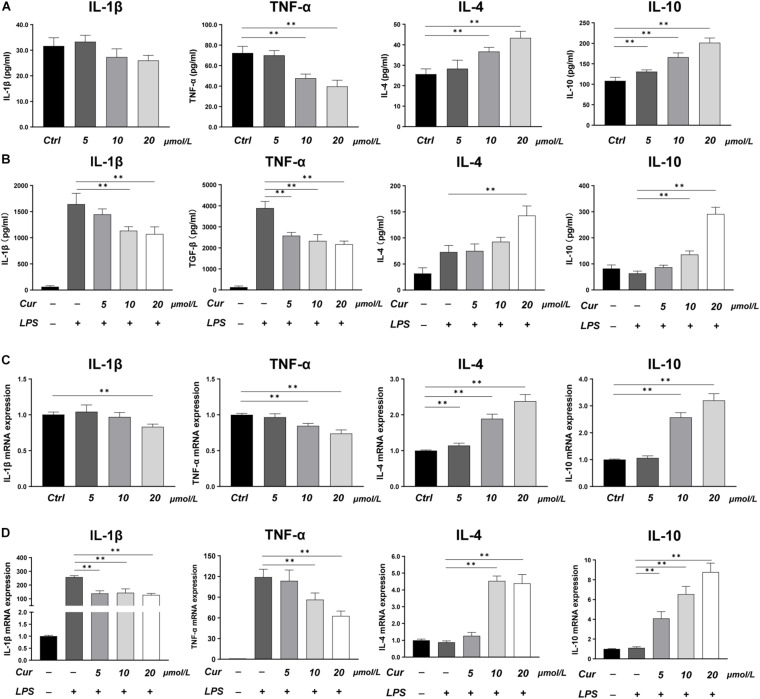
**(A,B)** Concentrations of IL-1β, TNF-α, IL-4, and IL-10 in the cell supernatant measured by ELISA. **(C,D)** Gene expressions of the pro-inflammatory cytokines IL-1β and TNF-α, and the anti-inflammatory cytokines IL-4 and IL-10 measured by real-time PCR. ^∗∗^*P* < 0.01.

In this study, LPS was applied to induce a pro-inflammatory condition to investigate the immunoregulatory effects of curcumin. The expression of inflammatory cytokines was evaluated by RT-PCR and ELISA, while the phenotype of macrophages was identified by immunofluorescence staining and flow cytometry. The ELISA results showed that, in the macrophage CM without LPS stimulation, the curcumin downregulated the production of M1-related factors TNF-α and upregulated the production of M2-related anti-inflammatory factors including IL-4 and IL-10, while the effects of curcumin on production of IL-1β were not remarkable (Figure 1A). In the LPS-induced pro-inflammation condition, the curcumin was found to have great anti-inflammatory capacity. The production of pro-inflammatory cytokines TNF-α and IL-1β was significantly increased, and this effect could be suppressed by curcumin in a dose-dependent way. Meanwhile, the expressions of IL-4 and IL-10 were remarkably upregulated by curcumin than that in the LPS group (Figure 1B). The RT-PCR results for IL-1β, TNF-α, IL-4, and IL-10 were consistent with the ELISA results and further investigated the effects of the curcumin on downregulating the expression of the pro-inflammatory factors TNF-α and IL-1β and increasing the anti-inflammatory factors IL-4 and IL-10 secretion (Figures 1C,D).

**FIGURE 2:**
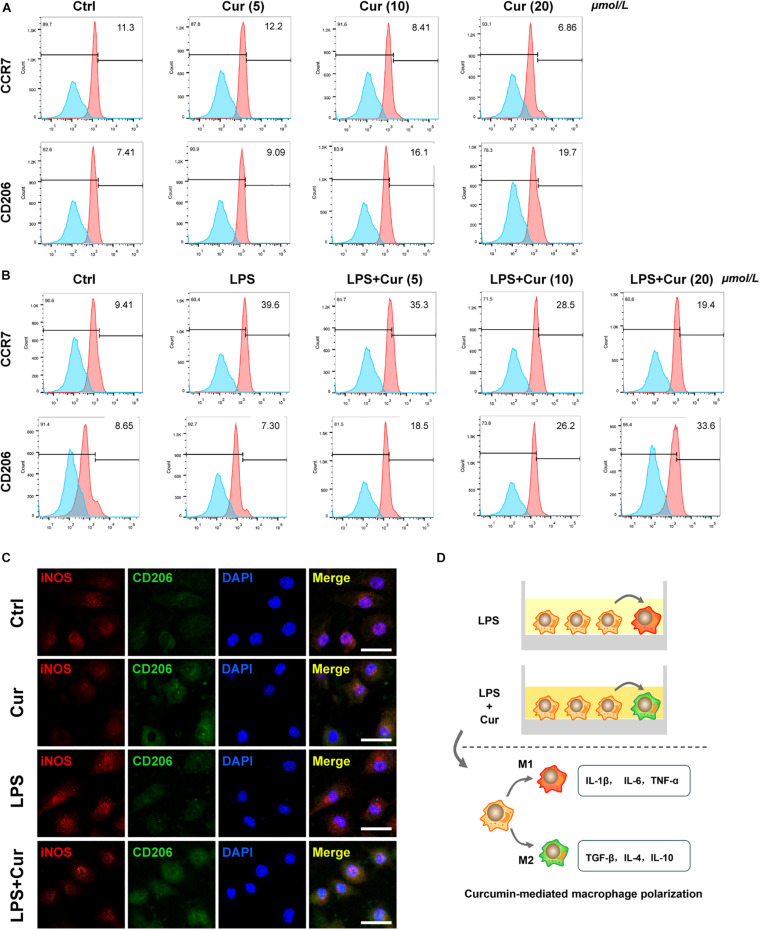
**(A,B)** Flow cytometry analysis of CCR7 and CD206 (numbers are the percentage of the cells’ population at respective groups). **(C)** Immunofluorescent staining of macrophages with iNOS (red), CD206 (green), and nuclei (blue). Cur: curcumin (20 μmol/L). Scale bar = 5 μm. **(D)** Schematic diagram of curcumin-mediated macrophage polarization.

Furthermore, flow cytometry was used to determine the expression of surface marker of M1-phenotype macrophages (CCR7) and M2-phenotype macrophages (CD206). The results revealed that, in the non-inflammatory condition, curcumin was able to downregulate the positive rate of CCR7 and upregulate that of CO206 (Figure 2A). When LPS existed, the curcumin was able to suppress the up-regulatory effect of LPS on CCR7 expression and upregulate the CD206 expression (Figure 2B). Immunofluorescent staining results also showed that the proportion of CD206^+^ M2 macrophages increased, whereas the macrophages expressed less M1 macrophage markers CCR7 with the modulation of curcumin in both non-inflammatory and inflammatory condition (Figure 2C). It was notable that the curcumin with 20 μmol/L showed the best performance in terms of modulating anti-inflammation and macrophage polarization in all the above experiments. Figure 2D showed the schematic diagram of curcumin-mediated macrophage polarization.

### The Effects of Curcumin on the Expression of BMP-2 and TGF-β in Macrophages

**FIGURE 3:**
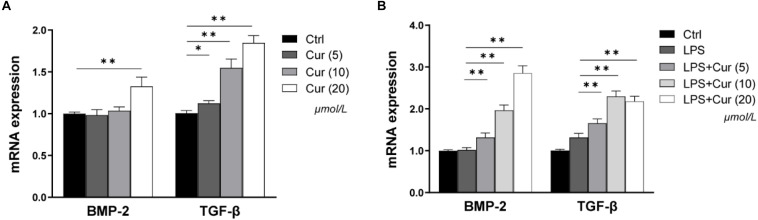
The gene expressions of BMP-2 and TGF-β in non-inflammatory environment **(A)** and inflammatory environment **(B)** measured by real-time PCR. ^∗^*P* < 0.05, ^∗∗^*P* < 0.01.

BMP-2 and TGF-β play important roles in the process of osteogenic differentiation of MSCs. Herein, in our study, the regulation of the two factors expression in macrophages by curcumin was detected *via* RT-PCR. As shown in Figure 3A, in the non-inflammatory condition, the BMP-2 and TGF-β mRNA level became significantly higher after treatment with 20 μmol/L curcumin compared with the Ctrl group, while the up-regulatory tendency of BMP-2 expression caused by 5 and 10 μmol/L curcumin was not significant (Figure 3A). In the inflammatory condition, the mRNA expression level of TGF-β was consistent. However, the effects of curcumin on BMP-2 became much more distinct. The curcumin in all experimental groups remarkably enhanced the mRNA expression of BMP-2 (Figure 3B).

### Osteogenic Differentiation of BMSCs Was Slightly Enhanced Under Curcumin-Modulated Inflammatory Condition

**FIGURE 4:**
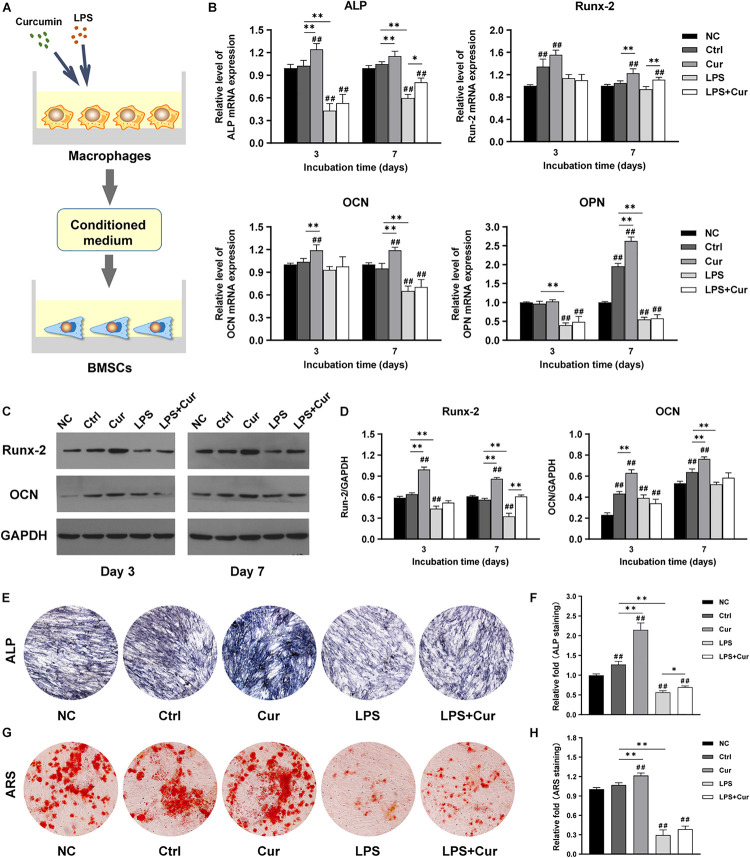
**(A)** Schematic illustration. **(B)** Relative mRNA expressions of osteogenic gene ALP, Runx-2, OCN, and OPN in BMSCs stimulated by different CM for 3 and 7 days. **(C)** Western blot results of Runx-2 and OCN expression in BMSCs on days 3 and 7. **(D)** Quantitation of the protein expression levels of Runx-2 and OCN. **(E,F)** ALP staining pictures and analysis of the BMSCs cultured in corresponding CM for 7 days. **(G,H)** ARS images and quantitative analysis of the BMSCs cultured with corresponding CM for 14 days. ##*P* < 0.01, compared with the NC group. ^∗^*P* < 0.05, ^∗∗^*P* < 0.01.

To further investigate how curcumin promotes the osteogenic differentiation of BMSCs in different macrophage CM, macrophages were cultured with curcumin for 3 days. Then the supernatants were collected and added to cells and the osteogenic differentiation in different conditions was analyzed. Figure 4A shows the schematic diagram of the cell processing method. In the non-inflammatory condition, the mRNA expression level of ALP and OCN were both increased in the curcumin-modulated macrophage CM on day 3 and day 7, while the mRNA expression level of Runx-2 and OPN were only enhanced on day 7, compared with the Ctrl group (Figure 4B). A similar trend was found in the protein expressions. Western blot revealed that the Runx2 and OCN expression in the Cur group was significantly enhanced compared with that in the Ctrl groups at both time points (Figures 4C,D). Moreover, the ALP and ARS staining results indicated that the curcumin-regulated macrophage improved the ALP expression (Figures 4E,F) and promoted the formation of calcium nodules (Figures 4G,H) in BMSCs. However, in LPS-stimulated inflammatory condition, the osteogenic differentiation of BMSCs was suppressed. The osteogenesis-related gene and protein expression of BMSCs was significantly decreased after 7-day incubation with LPS-activated macrophage CM (Figures 4B–D). Similar results were found in the ALP and ARS staining assay in LPS group (Figures 4E–H). Interestingly, BMSCs cultured in curcumin-modulated inflammation macrophage CM (LPS + Cur group) were detected with higher gene expression level of ALP and Runx-2 when compared with those in the LPS group. A similar trend was found in the ALP activity. Nevertheless, the ARS staining results showed that the modulatory effect of curcumin on macrophage-related inflammatory environment failed to improve the calcium nodules formation.

### Osteogenic Differentiation of BMSCs Was Slightly Enhanced When Curcumin Directly Activated the BMSCs

**FIGURE 5:**
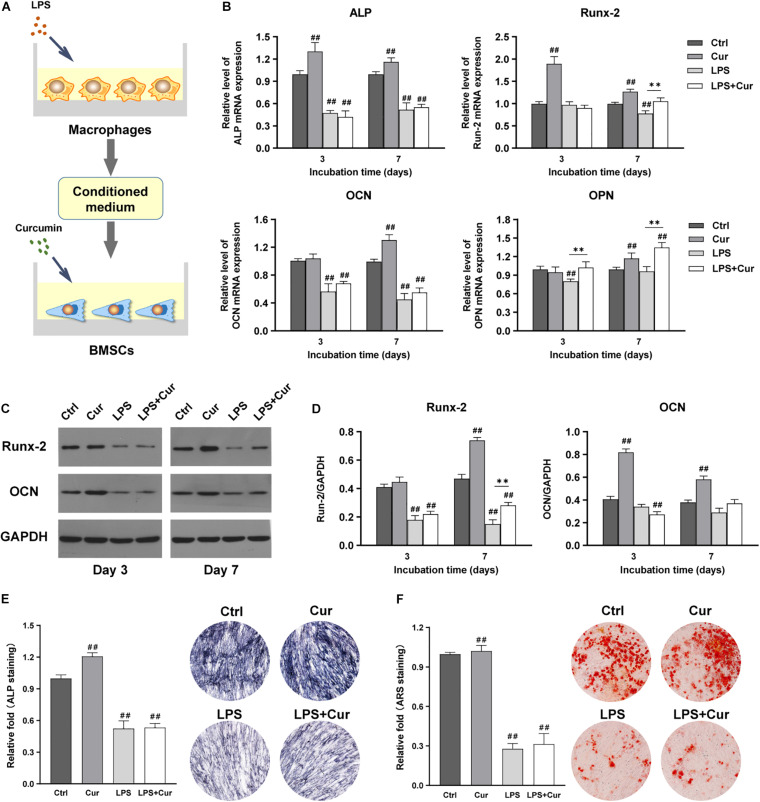
**(A)** Schematic illustration. **(B)** Relative mRNA expressions of osteogenic gene ALP, Runx-2, OCN, and OPN in BMSCs stimulated by different CM for 3 and 7 days. **(C)** Western blot results of Runx-2 and OCN expression in BMSCs on days 3 and 7. **(D)** Quantitation of the protein expression levels of Runx-2 and OCN. **(E)** ALP staining analysis and images of the BMSCs cultured in corresponding CM for 7 days. **(F)** ARS quantitative analysis and pictures of the BMSCs cultured with corresponding CM for 14 days. ##*P* < 0.01, compared with the NC group. ^∗∗^*P* < 0.01.

Figure 5A showed the schematic diagram of the cell processing method. PCR results revealed that the ALP, Runx-2, OCN, and OPN expression in Cur group were all remarkably increased at day 7 compared with Ctrl group (Figure 5B). Western blot showed the same trend in terms of the protein expression of Runx-2 and OCN (Figures 5C,D). In addition, ALP staining results showed that ALP activity of BMSCs in Cur group was much higher than those in Ctrl group (Figure 5E), while the difference of ARS staining between the two groups was not significant (Figure 5F). However, the directly modulatory effect of curcumin on BMSCs in the inflammatory microenvironment could only enhance the gene expression level of Runx-2 and OPN at day 7 compared with the LPS group. Moreover, the direct use of curcumin in BMSCs could not promote the ALP activity and the calcium nodule formation under inflammation environment. Therefore, the curcumin could promote the osteogenic differentiation of BMSCs through directly activating the BMSCs in non-inflammatory macrophage CM.

### Curcumin Significantly Enhanced the Osteogenic Differentiation Through Modulating Both Macrophage and BMSCs

**FIGURE 6:**
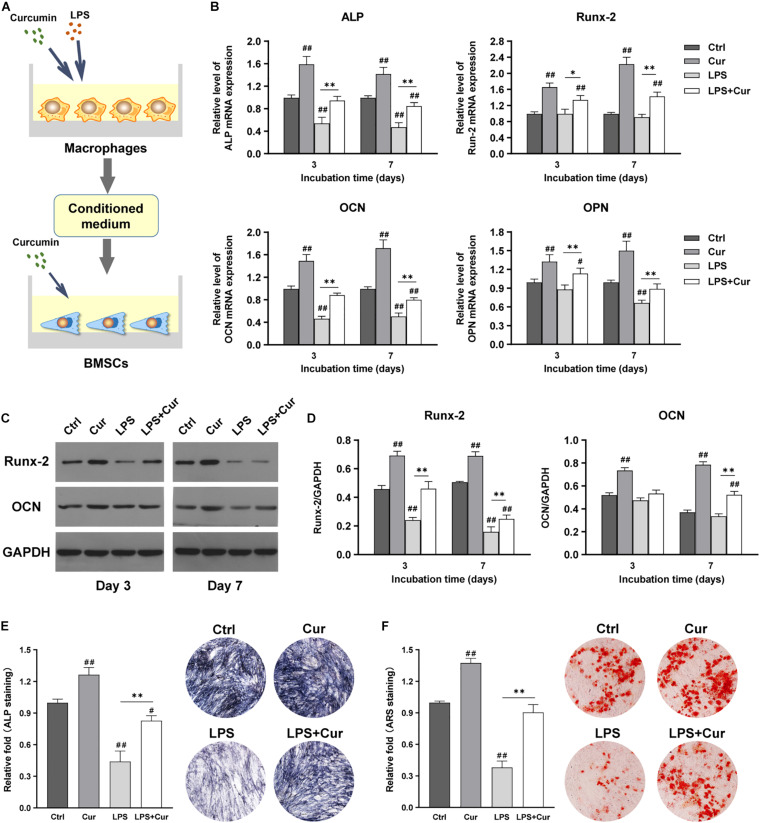
**(A)** Schematic illustration. **(B)** Relative mRNA expression of osteogenic gene ALP, Runx-2, OCN, and OPN in BMSCs stimulated by different CM for 3 and 7 days. **(C)** Western blot results of Runx-2 and OCN expression in BMSCs on days 3 and 7. **(D)** Quantitation of the protein expression levels of Runx-2 and OCN. **(E)** ALP staining analysis and pictures of the BMSCs cultured in different CM for 7 days. **(F)** ARS quantitative analysis and images of the BMSCs cultured with different CM for 14 days. ##*P* < 0.01, compared with the NC group. ^∗∗^*P* < 0.01.

Figure 6A showed the schematic diagram of the cell processing method. With the combination of the regulatory influence on both macrophages and BMSCs, the curcumin could remarkably enhance the mRNA expression levels of ALP, Runx-2, OCN, and OPN and protein expression levels of Runx-2 and OCN regardless in non-inflammatory or inflammatory conditions (Figures 6B–D). Moreover, the ALP activity results were consistent with the findings of PCR as the ALP staining intensity becomes much higher than LPS group (Figure 6E). More importantly, ARS staining showed that the size and quantity of the mineral nodules generated by BMSCs in curcumin-modulated inflammatory microenvironment were much larger than those in the LPS group (Figure 6F).

Taken together, these findings demonstrate that the curcumin could effectively promote osteogenic differentiation and mineralization through modulating the crosstalk between macrophages and BMSCs, indicating that the curcumin possessed great value and potential for the bone tissue injury treatment.

## Discussion

Bone tissue repair and regeneration after injuries involves a sequence of inflammatory responses and immunomodulation dominated by macrophages, leading to the recruitment and osteogenic differentiation of MSCs (Claes et al., 2012). In the process, the crosstalk between MSCs and macrophages is crucial for achieving optimal regeneration (Guder et al., 2020). However, the exact roles of macrophages in the bone regeneration process still remain unclear. Recently, the research has found the potential mechanisms underlying the interaction between macrophages and MSCs and provided innovative strategies to enhance bone regeneration by targeting the immunomodulation (Chen Z.T. et al., 2017; Shi et al., 2017; Zhang et al., 2018).

With this in mind, strategies to modulate macrophages activity to produce a desirable immune microenvironment thus promoting osteogenic differentiation of BMSCs are key points of this study. Recent research has reported that the functional components in certain Chinese herbs could influence macrophages activities and switch the phenotype from M1 to M2 (Yu et al., 2016; Okuda-Hanafusa et al., 2019). These small molecules were suitable for clinical application due to the convenience of synthesis, preservation, and standardization (Chen et al., 2016). Among the ingredients, curcumin has been shown to have anti-inflammatory and immunomodulatory properties in bone regeneration (Yang et al., 2020). Therefore, in the present study, we investigated the effects of curcumin on the immune response of macrophages and subsequently osteogenic differentiation of BMSCs under non-inflammatory or inflammatory condition.

The functional dynamic transformation of the macrophage phenotype plays crucial roles in inflammatory response and bone repair, but the most favorable phenotype for osteogenesis has not yet been confirmed (Chen et al., 2016). Generally, macrophages are divided into M1 and M2 phenotypes. The M1 macrophage is considered to induce proinflammatory activities by secreting pro-inflammatory cytokines such as TNF-α, IL-1β, and iNOS, which may lead to bone resorption and tissue destruction; while M2 macrophage, characterized by secreting anti-inflammatory cytokines such as IL-4, IL-10, and Arg-1, is believed to be more closely related to tissue repair and new bone formation (Gordon, 2003). Excessive M1 macrophage-induced inflammation is found to directly or indirectly cause the bone resorption and even destruction. According to these theories, a phenotype transition of the macrophages from M1 to M2 during bone repair process could be the innovative strategy for bone regeneration. Several researchers have suggested that modulation of macrophage phenotype from M1 to M2 would promote the recruitment of MSCs on the injury sites and their osteogenic differentiation (Spiller and Koh, 2017). In fact, curcumin have been reported to alleviate pro-inflammatory response of M1 macrophages and promote M2 macrophages polarization (Mohammadi et al., 2019). However, the effect of inflammatory microenvironment modulated by curcumin on osteogenic differentiation of MSCs remains unknown. In the present study, the LPS was used to induce the macrophages polarization into M1 phenotype with the increased secretion of pro-inflammatory factors TNF-α and IL-4. During this process, curcumin suppressed the pro-inflammatory phenotype polarization and increased the anti-inflammatory phenotypic switch. Thus, curcumin had great potential to create a favorable immune condition for tissue regeneration. In addition, at present, studies concerning the immunomodulatory effects of curcumin on macrophage-related immune microenvironment have drawn consistent conclusions. Zhang et al. indicated that curcumin significantly suppressed the LPS-induced neuroinflammation through promoting microglial M2 polarization. Similarly, Li B. et al. (2017) found that curcumin inhibited Ti particle-induced inflammatory response of macrophages by regulating macrophage polarization. Moreover, Li et al. (2021) fabricated a novel coating containing curcumin loaded nanocontainers on Mg-based implants and found the coatings remarkably modulated the macrophages polarization toward anti-inflammation phenotype and improved the immune microenvironment for tissue regeneration. Consistent with the aforementioned study, curcumin with 20 μmol/L was found to have better immunomodulatory effects to improve the inflammatory condition for bone healing through modulating inflammatory response, phenotype polarization of macrophages.

It has been confirmed that the macrophages-related immune microenvironment plays an important role in the process of osteogenic differentiation (Sima and Glogauer, 2013). M2 phenotype macrophages were found to release anti-inflammatory factors that promote the osteogenic differentiation in co-culture system (Salimuddin et al., 1999). In line with these data, we speculated that curcumin with immunomodulatory effects on macrophages might enhance the MSCs osteogenic differentiation through regulating the inflammatory microenvironment. Thus, in this study, the RAW 264.7 cells and BMSCs coculture system that allow the exchange of soluble factors without direct cell contact was established. Then the osteogenic differentiation of BMSCs was analyzed in a different macrophage conditioned medium. In the LPS-induced inflammatory microenvironment, the osteogenic differentiation of BMSCs was remarkably inhibited, which may be due to the pro-inflammatory factors in the CM released by macrophages polarized to an M1 phenotype. Similarly, other research suggested that pro-inflammatory factors such as TNF-α and IL-1β were able to inhibit osteogenic differentiation *in vitro* (Yang et al., 2013; Sullivan et al., 2014). Consistent with these data, an *in vivo* study demonstrated that TNF-α play an important role in LPS-induced inhibition of osteogenesis in a murine model (Gilbert et al., 2005). In the same way, we have demonstrated that the LPS-treated macrophage CM contained more TNF-α and IL-1β than that of the other groups, consequently leading to inhibition of osteogenic differentiation of BMSCs. Moreover, our study also reported that, in non-inflammatory condition, the osteogenic activities of BMSCs were enhanced in the CM regulated by curcumin on macrophages. However, in the inflammatory condition, even though the curcumin was able to decrease the release of pro-inflammatory factors and reverse the switch of M1 macrophage polarization in LPS condition, the osteogenic differentiation of BMSCs was only rejuvenated to some extent, and the long-term calcium nodule was hardly to be observed. These results indicated that curcumin did promote the osteogenesis both in non-inflammatory and inflammatory condition in a short term. However, in the long-term inflammatory condition, curcumin failed to improve the impaired osteogenic ability of BMSCs. So, in the macrophage-BMSCs coculture system, merely relying on inflammatory effects of curcumin on macrophages to modulate the immune condition might not be enough to reverse the long-term calcification formation.

Then we explored the individual influence of curcumin on BMSCs regardless of the regulation of macrophage. The osteogenic differentiation results of BMSCs were evaluated in the macrophage CM treated without or with LPS. Consistent with other studies, the curcumin was able to enhance the osteogenic differentiation of BMSCs in the non-inflammatory microenvironment (Son et al., 2018; Shi et al., 2020; Xiong et al., 2020). However, the enhancement of osteogenesis of BMSCs by curcumin was impaired a lot in the microenvironment with a strong inflammatory response. Thus, only focusing on the direct role of curcumin on BMSCs was still not enough to remarkably increase the osteogenic activity and long-term osteogenic potential of BMSCs in the M1 macrophage-related inflammatory microenvironment. Considering that an unfavorable immune microenvironment is detrimental to the capacity of bone cell differentiation, the regulation of the macrophage-mediated immune condition is quite crucial for successful bone regeneration.

Therefore, the above results suggest that the curcumin could promote the osteogenic differentiation through modulating the macrophage-related inflammation or directly modulating the BMSCs. In light of these, we speculated that the combination of the two interrelated and indispensable effects of curcumin together might enhance the bone regeneration to a great extent *in vitro*. As we expected, with the interaction between curcumin-modulated macrophages and curcumin-activated BMSCs, curcumin markedly enhanced the osteogenic differentiation of BMSCs and promoted the long-term calcium nodule formation in the inflammatory condition.

Above all, these results suggested that the curcumin facilitated the osteogenic differentiation through modulating the crosstalk between macrophages and BMSCs, indicating that curcumin might have great potential for bone regeneration. We provided only limited information about the complicated process of bone healing and indicated several molecules and cell types that might be involved in the process. Further studies are still needed to confirm the direct contribution of curcumin to immune modulation and subsequent osteogenic differentiation. Besides, the mechanisms behind the phenomenon also remained unclear. The efficacy of curcumin based on immunomodulation for bone tissue regeneration needs to be further investigated *in vivo*.

## Conclusion

The present study, for the first time, reported the effective role of curcumin on bone tissue regeneration *via* modulating the crosstalk between macrophages and BMSCs. We found that the curcumin was able to improve favorable immune microenvironment through regulating inflammatory response, macrophage polarization, and cytokines production, and sequentially enhance the osteogenic differentiation of BMSCs. These findings implied potential therapeutic applications of curcumin for bone tissue engineering and regeneration.

## Data Availability Statement

The original contributions presented in the study are included in the article/supplementary material, further inquiries can be directed to the corresponding author/s.

## Ethics Statement

All experimental protocols were approved by the Ethical Committee for Animal Experiments of The First Affiliated Hospital of Zhengzhou University.

## Author Contributions

SC, HLia, and HLiu designed the research. SC, HLia, and YW performed the experiments. HK, CZ, LY, and LL acquired and analyzed the data. SC, YJ, GS, CS, and ZS conceived the study and wrote the manuscript. The integrity of this work is guaranteed by SC and HLiu. All authors contributed to the article and approved the submitted version.

## Conflict of Interest

The authors declare that the research was conducted in the absence of any commercial or financial relationships that could be construed as a potential conflict of interest.
